# Diagnostic accuracy of a set of clinical and radiological criteria for screening of COVID-19 using RT-PCR as the reference standard

**DOI:** 10.1186/s12890-023-02369-9

**Published:** 2023-03-09

**Authors:** Mirella Cristine Oliveira, Karoleen Oswald Scharan, Bruna Isadora Thomés, Rafaella Stradiotto Bernardelli, Fernanda Baeumle Reese, Amanda Christina Kozesinski-Nakatani, Cintia Cristina Martins, Suzana Margareth Ajeje Lobo, Álvaro Réa-Neto

**Affiliations:** 1 Center for Studies and Research in Intensive Care Medicine – CEPETI, Monte Castelo Street, 366, Curitiba, Paraná 82590-300 Brazil; 2 Complexo Hospitalar do Trabalhador (CHT), República Argentina Street, 4406, Curitiba, Paraná 81050-000 Brazil; 3 School of Medicine and Life Sciences, Pontifical Catholic University of Paraná, Imaculada Conceição Street, 1155, Curitiba, Paraná 80215-901 Brazil; 4 Hospital Santa Casa de Curitiba, Praça Rui Barbosa, 694, Curitiba, Paraná 80010-030 Brazil; 5 Departament of Medicine, São José do Rio Preto Medical School, Brigadeiro Faria Lima avenue, 5416, São José do Rio Preto, São Paulo 15090-000 Brazil; 6 Internal Medicine Department, Hospital de Clínicas, Federal University of Paraná, General Carneiro Street, 181, Curitiba, Paraná 80060-900 Brazil

**Keywords:** COVID-19 testing, Intensive care units, Tomography, Sensitivity and specificity, COVID-19 testing, Mass screening

## Abstract

**Background:**

The gold-standard method for establishing a microbiological diagnosis of COVID-19 is reverse-transcriptase polymerase chain reaction (RT-PCR). This study aimed to evaluate the accuracy, sensitivity, specificity, positive predictive value (PPV), and negative predictive value (NPV) of a set of clinical-radiological criteria for COVID-19 screening in patients with severe acute respiratory failure (SARF) admitted to intensive care units (ICUs), using reverse-transcriptase polymerase chain reaction (RT-PCR) as the reference standard.

**Methods:**

Diagnostic accuracy study including a historical cohort of 1009 patients consecutively admitted to ICUs across six hospitals in Curitiba (Brazil) from March to September, 2020. The sample was stratified into groups by the strength of suspicion for COVID-19 (strong *versus* weak) using parameters based on three clinical and radiological (chest computed tomography) criteria. The diagnosis of COVID-19 was confirmed by RT-PCR (referent).

**Results:**

With respect to RT-PCR, the proposed criteria had 98.5% (95% confidence interval [95% CI] 97.5–99.5%) sensitivity, 70% (95% CI 65.8–74.2%) specificity, 85.5% (95% CI 83.4–87.7%) accuracy, PPV of 79.7% (95% CI 76.6–82.7%) and NPV of 97.6% (95% CI 95.9–99.2%). Similar performance was observed when evaluated in the subgroups of patients admitted with mild/moderate respiratory disfunction, and severe respiratory disfunction.

**Conclusion:**

The proposed set of clinical-radiological criteria were accurate in identifying patients with strong *versus* weak suspicion for COVID-19 and had high sensitivity and considerable specificity with respect to RT-PCR. These criteria may be useful for screening COVID-19 in patients presenting with SARF.

## Background

The SARS-CoV-2 emerged in late 2019 and has spread globally. The clinical presentation of patients infected with this virus may range from lack of symptoms to severe acute respiratory failure (SARF), culminating in admission to an intensive care unit (ICU) [1].

During a pandemic, viruses transmit at a high rate, infecting a large number of individuals and increasing the risk of disease, including in its most severe forms. Therefore, proper screening and knowledge of the clinical and epidemiological profile of infected individuals can contribute to timely actions and implementation of treatments that minimize the impact of the disease on individual and population levels [2]. Since the beginning of the health crisis due to COVID-19, the Brazilian health care system has faced challenges in discriminating patients with COVID-19 from those with other causes of SARF. Equally challenging has been the identification of patients who meet the criteria for severe disease. With the high demand and limited availability of beds, it is fundamental to properly guide those patients requiring high-level care to admission to ICUs instead of general medical wards.

The clinical manifestations of COVID-19 are also commonly found in several other diseases, *e.g.*, SARF due to other viruses, bacterial pulmonary infections, and decompensated chronic pulmonary or heart diseases, among others [3]. Therefore, establishing an accurate diagnosis is crucial, given the importance of each disease involving specific therapeutic decisions and, in the context of a pandemic, the need for the adoption of appropriate health measures.

The gold-standard method for establishing a microbiological diagnosis of COVID-19 is reverse-transcriptase polymerase chain reaction (RT-PCR). However, the low sensitivity of RT-PCR tests available at the beginning of the pandemic has resulted in a high number of false-negative results, a condition that has favored the community spread of the virus [2, 4, 5]. Also, this method is not widely available in Brazil, further limiting its availability considering the exponential spread of the disease—and even when the test is available, results may be delayed. These complications have also been reported in other (underdeveloped and developed) countries [6].

In this context, this study aimed to evaluate the accuracy, sensitivity, specificity, and positive and negative predictive values of a set of clinical-radiological criteria for screening of COVID-19 in patients with SARF admitted to ICUs, using RT-PCR as the reference standard for the diagnosis of the disease.

## Methods

Diagnostic accuracy study using data from a historical cohort of adult patients consecutively admitted to ICUs across six hospitals (two public and four private) in the city of Curitiba (Paraná, Brazil) due to SARF and suspected COVID-19 between March 11 and September 20, 2020.

The data were retrieved from the database of the Center for Studies and Research in Intensive Care Medicine (CEPETI), which encompasses sociodemographic and clinical information regarding patients' admission and outcome across all six ICUs included in the study. The study was approved by the ethics committee of the *Instituto de Neurologia de Curitiba* under protocol number 3.000.353 on November 05th, 2018 (ID: CAAE 98,099,918.2.0000.5227; project title: Epidemiological analysis of patients hospitalized in ICUs in Curitiba-Paraná). The requirement for informed consent was waived, given the noninterventional design of the study and the fact that the data were collected from clinical records and without contact with the participants. All research procedures were conducted in accordance with the ethical standards of the institutional committee on human experimentation and the Declaration of Helsinki of 1975, as revised in 2013. The study findings are reported according to STARD 2015 guidelines [7].

Data of all consecutive patients with suspected COVID-19 admitted to the participating ICUs with moderate to severe disease (as per the WHO Clinical Progression Scale) [8] during the study period were reviewed for identification of the level of suspicion (strong *versus* weak) of COVID-19 according to the proposed set of clinical-radiological criteria. Patients who fulfilled all three criteria listed below were classified as having strong suspicion for COVID-19:

1. Presence of one or more flu-like symptoms (fever, runny nose, sore throat, or cough).
2. Presence of two or more items in the quick Sequential Organ Failure Assessment (qSOFA) [9], *i.e.*, systolic blood pressure ≤ 100 mmHg, respiratory rate ≥ 22 bpm, reduced level of consciousness (Glasgow Coma Scale < 15), and oxygen pulse saturation ≤ 93%.
3. Chest computed tomography (CT) scanning obtained within the first 24 h of ICU admission and with a strong suspicion for COVID-19, defined as the presence of multifocal peripheral lesions distributed across both lungs and ground-glass infiltrates in more than 25% of the lung area, as described in the literature [10].

Patients not meeting all three criteria were classified as having weak suspicion for COVID-19. All patients were allocated into one of the two groups of suspicion for COVID-19 defined by the systematically applied set of clinical-radiological criteria (strong *versus* weak level of suspicion) and, within the first 24 h of admission, underwent nasopharyngeal swab collection for RT-PCR test for the detection of SARS-CoV-2.

We excluded from the analysis those patients without any of the three criteria described above or in whom the criteria were not evaluated and those with inconclusive or missing RT-PCR results.

The group of patients identified as having strong suspicion for COVID-19 was compared with the group with a weak suspicion for COVID-19 with respect to the following variables: age, sex, reduced level of consciousness according to the Glasgow Coma Scale, need for hemodynamic support with volume and/or vasoactive drug, need for invasive ventilatory support on ICU admission, Acute Physiology and Chronic Health Evaluation II (APACHE II) severity score, SOFA score in the first 24 h of ICU admission, reason for hospitalization, length of ICU stay, limitation of advanced life support, and mortality.

The accuracy of the screening method for COVID-19 based on the proposed set of clinical-radiological criteria (strong *versus* weak suspicion) was evaluated against the result of the RT-PCR (positive *versus* negative), which is the gold-standard method for diagnosis of COVID-19 [11].

The physicians analyzing the clinical-radiological criteria had no access to the results of laboratory tests confirming the diagnosis of COVID-19. Similarly, the laboratory team that ran the RT-PCR tests for detection of SARS-CoV-2 had no access to the patients' clinical data and were, thus, unaware of the patients' classification according to the clinical-radiological criteria.

### Statistical analysis

We included in the analysis only those patients with complete data regarding the parameters evaluated in the set of clinical-radiological criteria and with RT-PCR results for the detection of SARS-CoV-2. Therefore, imputation for missing data was not required.

To compare the diagnostic accuracy of the set of clinical-radiological criteria with the RT-PCR result (reference standard), we calculated the following measures of diagnostic accuracy: sensitivity, specificity, positive and negative predictive values, probability of false positive and false negative, accuracy (and respective 95% confidence intervals [95% CIs]), and likelihood values for positive and negative tests. The same analysis was also performed separately for the subgroup of patients with mild/moderate respiratory dysfunction and severe respiratory dysfunction, to assess the tool’s performance in different poles of respiratory dysfunction. In this context, patients with severe respiratory dysfunction were considered those admitted to the ICU using invasive or non-invasive mechanical ventilation, and/or those who scored three or more in the APACHE II respiratory parameters of the first 24 h in the ICU stay (respiratory rate ≥ 35 or < 5, arterial-alveolar gradient ≥ 350 mmHg in inspired oxygen fraction ≥ 0.5, or oxygen partial pressure ≤ 60 mmHg in inspired oxygen fraction < 0.5). The others were considered as mild/moderate respiratory dysfunction.

As an exploratory analysis, for those patients with results that were true negative, false positive, and false negative according to the set of clinical-radiological criteria, we grouped the reasons for hospitalization (defined up to 24 h after admission) and described them using absolute frequencies and percentages.

The association of dichotomous variables (sex, mortality, requirement of hemodynamic and/or invasive ventilatory support at ICU admission) between the groups (strong *versus* weak suspicion) was presented as absolute and percentage frequencies and compared using Fisher's exact test. The results of the Glasgow Coma Scale on admission were dichotomized, and scores ≤ 14 indicated a reduced level of consciousness while a score of 15 indicated a normal level of consciousness; these variables were described as absolute frequencies and percentages, and the differences between groups were assessed using Fisher's exact test.

The normality of the distribution was verified visually using box plots, with skewness and kurtosis assessments, and with the Kolmogorov–Smirnov test. The age of the patients, given their normal distribution, was described as means and standard deviations and compared between groups using Student's *t* test for independent samples. The length of ICU stay, which was not normally distributed, and APACHE II and SOFA scores and the Glasgow Coma Scale were described as medians and interquartile ranges and compared between groups using the nonparametric Mann–Whitney test. For the evaluation of the influence of the APACHE II score on the proposed set of clinical-radiological criteria, the scores were further adjusted for age, analyzed using a multiple logistic regression model, and presented as odds ratios (ORs) and 95% confidence intervals.

The level of statistical significance was set at 5%, and the data were analyzed using Stata, version 15.0 (StataCorp LLC, College Station, TX, USA).

## Results

**Fig. 1:**
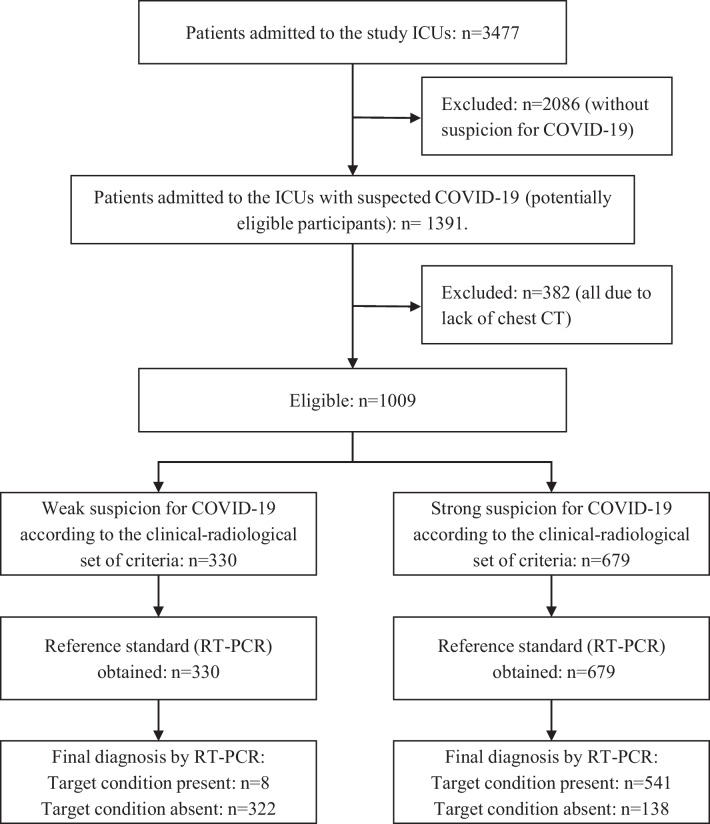
Flowchart with the process of selection of the study sample. Abbreviations: ICU, intensive care unit; CT, Computed tomography; RT-PCR, Reverse transcription polymerase chain reaction

During the study period, 3477 patients were admitted to the participating ICUs, of whom 1391 were suspected of having COVID-19. Of these, 382 were excluded as they had not been evaluated with chest CT scanning, resulting in 1009 eligible patients who fulfilled all the inclusion criteria (Fig. 1).

Of the 1009 patients included in the analysis, 679 (67.3%) and 330 (32.7%) were identified as having, respectively, a strong and weak suspicion for COVID-19 according to the set of clinical-radiological criteria. Table 1 shows the clinical characteristics associated with hospitalization and outcomes in each group.

**Table 1:** Characteristics of ICU patients with SARF and suspicion for COVID-19, stratified by the clinical-radiological criteria

Characteristics	Total (n = 1009)	Strong Suspicion (n = 679)	Weak Suspicion (n = 330)	*p value*
Age (years)—mean (SD)	63.4 (17.5)	61.5 (16.5)	67.1 (18.2)	≤ 0.001^f^
Male sex—n (%)	554 (54.9)	392 (57.7)	162 (49.1)	0.010^g^
*Clinical condition at ICU admission*				
Reduced level of consciousness—n (%) ^a^	299 (32.6)	175 (28.8)	124 (39.9)	≤ 0.001^g^
Need for hemodynamic support—n (%) ^a^	257 (28)	165 (27.2)	92 (29.6)	0.485^g^
Need for invasive ventilatory support—n (%) ^a^	176 (19.2)	104 (17.1)	72 (23.2)	0.033^g^
*Severity scores at ICU admission*				
APACHE II score—median (IQR) ^b^	12 (7–18)	11 (6–17)	13 (9–18)	≤ 0.001^h^
SOFA score – median (IQR) ^c^	3 (2–6)	3 (2–6)	3 (2–6)	0.365^h^
Severe respiratory disfunction at ICU admission—n (%) ^d^	468 (46.4)	318 (46.8)	150 (45.5)	0.687^g^
*Outcome*				
Length of ICU stay (days)—median (IQR)	4.0 (1.9–8.3)	4.5 (2.2–9.3)	3.1 (1.3–5.7)	≤ 0.001^h^
Limitation of life-sustaining treatments (no increment or withdrawal)—n (%) ^e^	66 (6.6)	43 (6.3)	23 (7)	0.685^g^
Death—n (%)	246 (24.4)	191 (28.1)	55 (16.7)	≤ 0.001^g^

Clinical characteristics associated with hospitalization and outcomes in patients admitted to intensive care units with severe acute respiratory failure and suspicion for COVID-19, stratified by the strength of the suspicion according to the proposed set of clinical-radiological criteria

*ICU* intensive care unit, *APACHE II* Acute Physiology and Chronic Health disease Classification System II, *SOFA* Sequential Organ Failure Assessment, *SD* standard deviation, *IQR* interquartile range

^a^91 missing data in the total sample, 71 in the strong suspicion group, and 19 in the weak suspicion group

^b^79 missing data in the total sample, all in the strong suspicion group

^c^428 missing data in the total sample, 316 in the strong suspicion group, and 112 in the weak suspicion group

^d^patients with severe respiratory dysfunction were considered those admitted to the ICU using invasive or non-invasive mechanical ventilation, and/or those who scored three or more in the APACHE II respiratory parameters of the first 24 h in the ICU stay (respiratory rate ≥ 35 or < 5, arterial-alveolar gradient ≥ 350 mmHg in inspired oxygen fraction ≥ 0.5, or oxygen partial pressure ≤ 60 mmHg in inspired oxygen fraction < 0.5. The others were considered as mild/moderate respiratory dysfunction)

^e^Three patients who progressed with brain death were not considered in the analysis, including one who was in the strong suspicion group and two in the weak suspicion group

^f^Significance of Student's *t* test for independent samples

^g^Significance of Fischer's exact test

^h^Significance of nonparametric Mann–Whitney test

The group of patients with a strong versus that with a weak suspicion for COVID-19 had more men and individuals older than 60 years, were less frequently identified as having reduced level of consciousness or requiring invasive ventilatory support at ICU admission. This group of patients also had a lower median APACHE II score. However, after adjustment of the APACHE II scores by age, the difference between groups was no longer significant (OR 0.993, 95% CI 0.976–1.01). The median length of ICU stay and the percentage of deaths were also higher in the strong *versus* weak suspicion group.

The time from symptom onset to ICU admission among patients with a positive RT-PCR result was 6.8 ± 4.3 days. Table 2 shows the results of the classification by the proposed set of clinical-radiological criteria compared with the results of the RT-PCR tests.

**Table 2:** Cross tabulation of the results of the proposed set of clinical-radiological criteria versus the RT-PCR, considering the total sample

Total sample		RT-PCR (reference standard)			
		Positive	Negative	Total	
Classification by the clinical-radiological criteria	Strong Suspicion	541 (53.6%)	138 (13.7%)	679 (67.3%)	PPV^a^ 79.7% (76.6–82.7%)
	Weak Suspicion	8 (0.8%)	322 (31.9%)	330 (32.7%)	NPV^a^ 97.6% (95.9–99.2%)
	Total	549 (54.4%)	460 (45.6%)	1009 (100%)	
		Sensitivity^a^ 98.5% (97.5–99.5%)	Specificity^a^ 70% (65.8–74.2%)		Accuracy^a^ 85.5% (83.4–87.7%)

*RT-PCR* Reverse transcription polymerase chain reaction, *PPV* positive predictive value, *NPV* negative predictive value

^a^Values presented with their respective 95% confidence intervals

Likelihood of a positive test: 3.28; likelihood of a negative test: 0.02

The proposed set of clinical-radiological criteria had high sensitivity (98.5%, 95% CI 97.5–99.5%) and comparatively lower specificity (70%, 95% CI 65.8–74.2%) to identify patients with a diagnosis of COVID-19 confirmed by RT-PCR. The probabilities of a false-positive and a false-negative result by the set of criteria were 30% (95% CI 25.8–34.2%) and 1.5% (95% CI 0.5–2.5%), respectively, and the positive and negative predictive values were 79.7% (95% CI 76.6–82.7%) and 97.6% (95% CI 95.9–99.2%), respectively. The proposed set of criteria had an accuracy of 85.5% (95% CI 83.4–87.7%) (Table 2). Similar performance of the set of clinical-radiological criteria was observed when evaluated in the subgroups of patients hospitalized with mild/moderate respiratory disfunction (Table 3) and severe respiratory disfunction (Table 4).

**Table 3:** Cross tabulation of the results of the proposed set of clinical-radiological criteria versus the RT-PCR, considering the subgroup of patients with mild/moderate respiratory disfunction at ICU admission

Subgroup of patients with mild/moderate respiratory disfunction		RT-PCR (reference standard)			
		Positive	Negative	Total	
Classification by the clinical-radiological criteria	Strong Suspicion	295 (54.5%)	66 (12.2%)	361 (66.7%)	PPV^a^ 81.7% (77.7–85.7%)
	Weak Suspicion	5 (0.9%)	175 (32.3%)	180 (33.3%)	NPV^a^ 97.2% (94.8–99.6%)
	Total	300 (55.5%)	241 (44.5%)	541 (100%)	
		Sensitivity 98.3% (96.9–99.8%)	Specificity^a^ 72.6% (67–78.2%)		Accuracy^a^ 86.9% (84–89.7%)

*RT-PCR* Reverse transcription polymerase chain reaction, *PPV* positive predictive value, *NPV* negative predictive value

^a^Values presented with their respective 95% confidence intervals

Likelihood of a positive test: 3.59; likelihood of a negative test: 0.02

**Table 4:** Cross tabulation of the results of the proposed set of clinical-radiological criteria versus the RT-PCR, considering the subgroup of patients with severe respiratory disfunction at ICU admission

Subgroup of patients with severe respiratory disfunction		RT-PCR (reference standard)			
		Positive	Negative	Total	
Classification by the clinical-radiological criteria	Strong Suspicion	246 (52.6%)	72 (15.4%)	318 (67.9%)	PPV^a^ 77.4% (72.8–82%)
	Weak Suspicion	3 (0.6%)	147 (31.4%)	150 (32.1%)	NPV^a^ 98% (95.8–99.9%)
	Total	249 (53.2%)	219 (46.8%)	468 (100%)	
		Sensitivity^a^ 98.8% (97.4–99.9%)	Specificity^a^ 67.1% (60.9–73.3%)		Accuracy^a^ 84.0% (80.7–87.3%)

*RT-PCR* Reverse transcription polymerase chain reaction, *PPV* positive predictive value, *NPV* negative predictive value

^a^Values presented with their respective 95% confidence intervals

Likelihood of a positive test: 3.01; likelihood of a negative test: 0.02

The proportion of confirmed COVID-19 diagnoses by RT-PCR was also not significantly different between those with mild/moderate respiratory disfunction (55.5% positive RT-PCR), and severe respiratory disfunction (53.2% positive RT-PCR) (*p* = 0.486).

Table 5 shows the reasons for ICU admission among patients with false-positive and false-negative results, as well as those patients with a true negative diagnosis, which can lead to diagnoses other than COVID-19.

**Table 5:** Frequency of reasons for hospitalization according to the proposed set of clinical-radiological criteria

Reasons for ICU admission, defined up to 24 h from admission	True negative n = 322	False positive n = 138	False negative n = 8
Pulmonary disease—n (%)	249 (77.3^a^)	130 (94.2^a^)	4 (50^a^)
Pneumonia or sepsis of pulmonary source—n (%)	145 (58.2^b^)	10 (76.8^b^)	2 (25^b^)
COPD—n (%)	48 (19.3^b^)	7 (5.4^b^)	0 (0^b^)
Influenza—n (%)	14 (5.6^b^)	2 (1.5^b^)	0 (0^b^)
SARF—n (%)^c^	41 (16.5^b^)	21 (16.2^b^)	2 (25^b^)
Tuberculosis—n (%)	1 (0.4^b^)	0 (0^b^)	0 (0^b^)
Heart disease—n (%)^d^	43 (13.4^a^)	5 (3.6^a^)	2 (25^a^)
Extrapulmonary sepsis with acute lung injury—n (%)	30 (9.3^a^)	3 (2.2^a^)	2 (25^a^)

Frequency of reasons for hospitalization of patients with false-positive and false-negative results and patients with a true negative diagnosis according to the proposed set of clinical-radiological criteria

*n* absolute frequency, % percentage, *SARF* severe acute respiratory syndrome, *ICU* intensive care unit

^a^Percentage considering the total number of cases for each column

^b^Percentage considering the total pulmonary reasons for each column

^c^Respiratory failure due to causes other than COVID-19

^d^All patients had congestive heart failure

## Discussion

The demographic and clinical characteristics of the study sample, which comprised patients admitted to ICUs with suspected COVID-19, were similar to those described in studies conducted in other locations severely affected by the pandemic [12–16]. As in this study, others also have sought to improve the screening process for COVID-19 by analyzing the relationship between chest CT findings and RT-PCR results in patients with suspected COVID-19, developing an artificial neural network, using logistic regression or proposing decision trees, in an attempt to diagnose the disease [2, 4, 17–20].

The findings of the present study showed a close relationship between the classification of strong suspicion for COVID-19 by the proposed set of clinical-radiological criteria and a positive RT-PCR result, with high sensitivity (98.5%) and a narrow confidence interval, suggesting that the diagnosis of COVID-19 is very unlikely in the absence of the proposed criteria. Thus, the high probability of a patient classified as having a strong suspicion for COVID-19 presenting a positive RT-PCR result suggests that the systematic application of the proposed set of criteria would have a positive impact on the screening of cases with suspected disease. This would translate into a more assertive approach in the provided care, including the application of appropriate sanitary measures and the implementation of specific treatment protocols even before the microbiological confirmation of the disease.

The high sensitivity of the proposed criteria may be related to the average duration of symptoms in the study population, which was relatively high, and the presence of tomographic changes [21], as chest CT findings may not be present in patients with a short symptom duration [22–24]. However, some clinical conditions at admission became confounders and led to a strong suspicion for the disease, which was not confirmed by microbiological examination. Among these confounding diseases are those that also present with SARF, such as acute exacerbations of chronic obstructive pulmonary disease, bacterial pneumonia, and extrapulmonary sepsis with acute pulmonary injury.

On the other hand, the high sensitivity encountered brought along a high percentage of false-positive results. However, it should be noted that RT-PCR has a high rate of false-negative results for the diagnosis of COVID-19, especially in more advanced stages of the disease; therefore, patients who are strongly suspected of having COVID-19 but have a negative RT-PCR result could have a positive result in a repeat RT-PCR test, so the possibility of the disease cannot be ruled out [10].

Our set of clinical-radiological criteria developed by intensive care physicians in March 2020—when there was still no complete knowledge about the signs and symptoms that could be related to the disease—based on the literature and used pragmatically to screen patients with SARF on ICU admission during the COVID-19 pandemic, showed sensitivity, specificity and accuracy values similar to those of diagnostic model for COVID-19 developed using logistic regression models and artificial neural network [18, 20].

We included in the set of clinical-radiological criteria respiratory symptoms and fever, which at the beginning of the pandemic were the most related to covid-19. This could explain the burden of other non-COVID-19 lung diseases in the false positive group, as many lung diseases share similar symptoms. However, other studies demonstrate that fever and respiratory symptoms, such as cough and rhinorrhea are the most strongly correlated with the diagnosis of COVID-19, although several other extrapulmonary signs and symptoms may be present in patients with COVID-19 [18, 19].

Among the patients identified as having a weak suspicion for COVID-19, we found only 1.5% of false-negative results; the confounders in these cases were other lung diseases, especially bacterial pneumonia.

The limitations of our study include the involvement of multiple intensivists in routinely evaluating the parameters of the clinical-radiological criteria and CT scans, which could have resulted in diverse interpretations of the findings, and the final reports of chest CTs being signed by different radiologists. As a strength, the group of professionals who performed the initial evaluation was small. Finally, we want to emphasize that all patients analyzed in this study had moderate to severe disease as determined by the WHO Clinical Progression Scale, *i.e.*, a score ≥ 5 in the 10-point table [8].

Finally, we consider that the study contributes with relevant information by presenting the possibility of screening patients with suspected COVID 19 based on clinical and radiological criteria without the use of RT-PCR, which was not always available, especially in low-income countries.

## Conclusion

The application of the criteria based on three clinical and radiological parameters proposed in this study could accurately identify patients with COVID-19 while RT-PCR results are pending. These criteria had high sensitivity and negative predictive value, and still considerable specificity and positive predictive value for a COVID-19 diagnosis when compared with the reference standard RT-PCR, in patients with mild/moderate respiratory dysfunction, as well as with severe respiratory dysfunction at ICU admission.

The adoption of these criteria may contribute to the timely implementation of recommended treatments in advance of the microbiological result and the application of sanitary actions such as placing patients in respiratory isolation and separating cohorts of inpatients with strong *versus* weak suspicion of COVID-19, thus helping the clinical management of these patients and reducing cross-contamination.

## Data Availability

The dataset supporting the conclusions of this article is available in the Zenodo repository, DOI 10.5281/zenodo.7140845 and hyperlink to dataset is 10.5281/zenodo.7140845.

## Abbreviations

RT-PCR: Reverse-transcriptase polymerase chain reaction
PPV: Positive predictive value
NPV: Negative predictive value
SARF: Severe acute respiratory failure
ICUs: Intensive care units
CI: Confidence interval
CEPETI: Center for Studies and Research in Intensive Care Medicine
qSOFA: Quick Sequential Organ Failure Assessment
CT: Computed tomography
APACHE II: Acute Physiology and Chronic Health Evaluation II
ORs: Odds ratios
SD: Standard deviation
IQR: Interquartile range
